# 279. Maternal Infection by *SARS CoV2* and Clinical Repercussion of the Newborn.

**DOI:** 10.1093/ofid/ofac492.357

**Published:** 2022-12-15

**Authors:** Maria C Casillas Casillas, Claudia C Lopez-Enriquez, Tania Salcido Madrid

**Affiliations:** Hospital Espanol, Ciudad de Mexico, Distrito Federal, Mexico; HOSPITAL ESPAÑOL, Ciudad de Mexico, Distrito Federal, Mexico; Hospital Espanol de Mexico, Ciudad de Mexico, Distrito Federal, Mexico

## Abstract

**Background:**

Pregnancy confers a higher risk of complications in SARS CoV2 disease as well as in the neonatal stage. Physiological and immunological changes during pregnancy predispose women to have complications in all the respiratory variations and increase maternal-fetal mortality and morbidity; The role of SARS CoV2 infection in newborns of mothers positive for COVID-19 was evaluated, analyzing the clinical consequences in the pregnant patient and the impact on the newborn.

**Methods:**

Descriptive, observational, retrospective study. All pregnant patients with a positive PCR test for SARS CoV2 and newborns treated at the Spanish Hospital, in the period from March 2020 to March 2021, were included, the information provided was collected from the medical history.

**Results:**

We recorded the cases of pregnant patients treated at the Spanish Hospital in the period from March 2020 to March 2021. The population was divided into those with a positive and negative PCR test for SARS Cov2. A total of 561 cases were registered, 35 positive cases for SARS Cov2; positive patients reported a higher presentation of Preeclampsia than patients without infection (p 0.009). The percentage of preterm products in mothers with infection was higher by 20% vs 12%; with the highest number of advanced neonatal resuscitation events. SARS Cov2 infection conditions the presentation of pathologies such as preeclampsia in pregnant women and an increase in preterm birth.

**Conclusion:**

In this study we conclude, comparing with the group of healthy pregnant women with no history of COVID-19, that the products of mothers with a history of SARS CoV2 infection represent a higher risk of prematurity coupled with the risk of other pathologies such as preeclampsia, also in the period neonatal patients required greater support with oxygen, however the rate of severe complications in the newborn is unlikely. The role of maternal protection such as isolation, hygiene and vaccination measures are priority tools to reduce these perinatal pathologies and the risk of contagion.

**Disclosures:**

**All Authors**: No reported disclosures.

